# Cutaneous toxicities secondary to STAT3 inhibition: A retrospective case series

**DOI:** 10.1016/j.jdcr.2025.03.027

**Published:** 2025-04-17

**Authors:** Kevin P. Lee, Apostolia M. Tsimberidou, Abhijit Chakraborty, Anisha B. Patel

**Affiliations:** a Department of Dermatology, The University of Texas MD Anderson Cancer Center, Houston, Texas; b Department of Investigational Cancer Therapeutics, The University of Texas MD Anderson Cancer Center, Houston, Texas

**Keywords:** cutaneous toxicity, immunotherapy toxicity, oncodermatology, signal transducer and activator of transcription protein, STAT3 inhibitor

## Introduction

Signal transducer and activator of transcription (STAT) 3 protein is 1 of a family of 7 STAT proteins that play important roles in cytokine and growth factor signaling.^1^ STAT3 signaling is hyperactive in many human cancers and associated with a worse prognosis.^2^^,^^3^ In tumor cells, STAT3 overexpression leads to immunosuppression by inhibiting innate and adaptive immune responses. In innate immunity, STAT3 inhibits many pro-inflammatory cytokines, interferons, and chemokines. However, increased STAT3 expression in adaptive immunity inhibits the function of effector T cells and their antitumor effects.^4^^,^^5^ STAT3 is the main mediator of the interleukin-6 signaling pathway and a major transcription factor in the differentiation of T helper 17 cells. Inhibition of STAT3 has been shown in animal studies to be effective for various autoimmune diseases, including psoriasis, rheumatoid arthritis, and inflammatory bowel disease.^6^ A topical ointment containing STA-21, a small molecule inhibitor of STAT3, improved psoriatic lesions in 6 out of 8 patients after 2 weeks of use.^7^ In addition to immunosuppression, STAT3 hyperactivation plays an important role in metastasis, antiapoptosis, and angiogenesis in tumors.^8^ Therefore, there has been an emerging focus on developing STAT3 inhibitors for oncologic therapy in solid tumors. We report a series of 8 patients with seemingly paradoxical reactions who developed cutaneous skin eruptions secondary to STAT3 inhibition.

## Methods

The institutional electronic medical record was queried for patients who received treatment on a clinical trial with a STAT3 inhibitor between January 2015 and October 2023 at MD Anderson Cancer Center and had at least a 6-month follow-up period from the time of drug initiation. A manual chart review was performed to evaluate for potential cutaneous toxicities.

## Results

A total of 52 patients treated with a STAT3 inhibitor were identified. 8 (15.4%) of these 52 patients developed cutaneous reactions, including 5 patients who were seen by a board-certified dermatologist and were determined to have cutaneous reactions secondary to STAT3 inhibitors based on the timeline and clinical presentation of the reaction. 3 additional patients (patients 4-6) had cutaneous reactions related to STAT3 inhibition determined by medical record chart review, and the association of the skin reaction with the STAT3 inhibitor was made by the treating physician on the basis of timing of the STAT3 inhibitor treatment and clinical presentation. Demographics and rash characteristics can be seen in Tables I and II, respectively.

**Table I:** Demographics of patients with cutaneous toxicities to STAT3 inhibitors

Patient no.	Age (y)	Gender	Cancer	Stage of cancer	Treatment length	Still on treatment	Reason for discontinuation	Concomitant medications	Liver metastases	Outcomes	Duration of disease
1	75	Female	HCC	4	63 d	No	Progression of disease	Amlodipine, vitamin D3, ibandronate, levothyroxine, oxycodone, allopurinol	Yes	Alive	70 mo
2	74	Male	HCC	4	10 d	No	Discontinued due to cutaneous reaction	Amlodipine, semaglutide	Yes	Alive	43 mo
3	66	Male	HCC	4	92 d	No	Progression of disease	Entecavir	Yes	Dead	28 mo
4	56	Male	HCC	4	13 d	No	Progression of disease	Levothyroxine, lisinopril, lorazepam, megestrol, ondansetron, pantoprazole, tramadol, ondansetron	Yes	Dead	14 mo
5	74	Male	HCC	4	70 d	No	Progression of disease	Magnesium, hydrochlorothiazide, lisinopril, furosemide, allopurinol, omeprazole, loratadine	Yes	Alive	176 mo
6	58	Male	HCC	4	84 d	No	Progression of disease	Allopurinol, amlodipine, alprazolam, vitamin B12, esomeprazole, lisinopril, tramadol, trazodone	Yes	Dead	48 mo
7	55	Female	Breast cancer	4	55 d	No	Progression of disease	Palbociclib, alprazolam, celecoxib, gabapentin, hydromorphone, methadone, letrozole	Yes	Alive	313 mo
8	46	Male	Melanoma	4	Ongoing (521 d)	Yes	N/A	Apixaban, diphenoxylate-atropine, loperamide, doxycycline, gabapentin, hydroxyzine, ibuprofen, levothyroxine, ondansetron	Yes	Alive	102 mo

*HCC*, Hepatocellular carcinoma; *STAT3*, signal transducer and activator of transcription 3.

**Table II:** Characteristics and treatment of cutaneous toxicities to STAT3 inhibition

	Time from initiation of STAT3 inhibitor to rash development	Seen by dermatologist	Rash type-clinically	Biopsy performed	Biopsy results	Treatment	Follow-up
Patient 1	10 d	Yes	Grade 3 Morbilliform	Yes	Sparse perivascular lymphohistiocytic infiltrate	Oral methylprednisolone and triamcinolone cream	Resolved at 19 d
Patient 2	10 d	Yes	Grade 3 Morbilliform	No	N/A	Triamcinolone cream	Resolved at 11 d
Patient 3	83 d	Yes	Leukodermic	No	N/A	None	Not resolved
Patient 4	8 d	No	Grade 1 Morbilliform	No	N/A	Triamcinolone cream	Resolved at 5 d
Patient 5	56 d	No	Grade 1 Unknown	No	N/A	Triamcinolone cream	Not resolved at 14 d
Patient 6	66 d	No	Grade 2 Unknown	No	N/A	Triamcinolone cream	Not resolved at 2 mo
Patient 7	83 d	Yes	Psoriasiform	No	N/A	Triamcinolone ointment and fluocinonide solution	Resolved at 30 d
Patient 8	25 d	Yes	Grade 3 Lichenoid	Yes	Lichenoid inflammatory infiltrate	Acitretin, betamethasone cream, and desonide ointment	Resolved at 18 mo

*STAT3*, Signal transducer and activator of transcription 3.

### Patient 1

**Fig 1:**
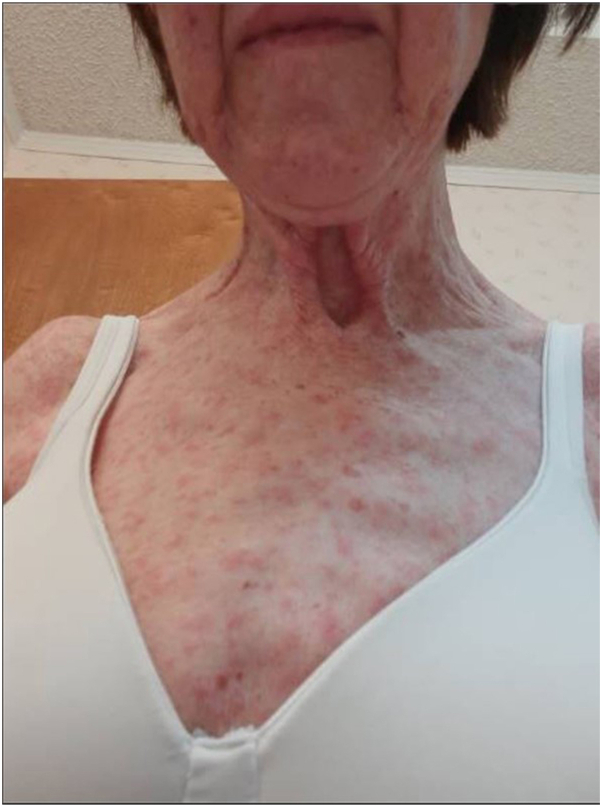
Erythematous papules on the upper chest and extremities of patient 1.

**Fig 2:**
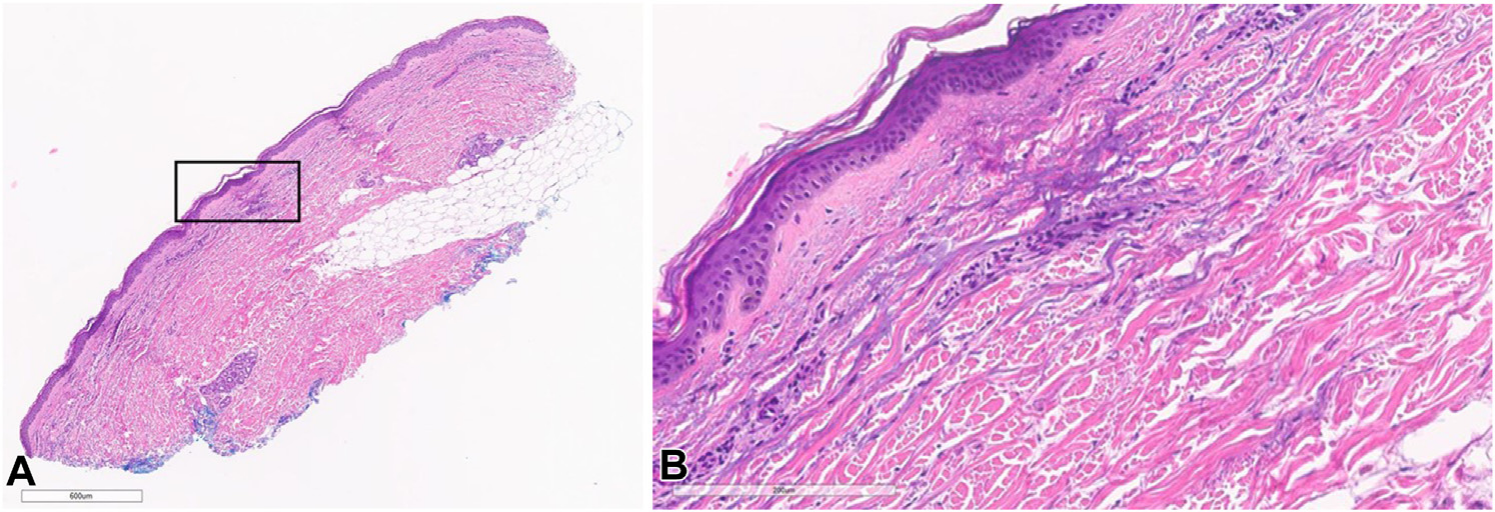
Hematoxylin-eosin stains showing perivascular lymphohistiocytic infiltrate from patient 1. **A,** Original magnification: ×2. **B,** Original magnification: ×20.

A 75-year-old female with metastatic hepatocellular carcinoma (HCC) developed a grade 3 morbilliform eruption on her trunk and extremities 10 days after starting STAT3 inhibitor treatment (Fig 1). The patient was seen by dermatology, and a punch biopsy from her right upper arm revealed a superficial perivascular lymphohistiocytic infiltrate (Fig 2, *A* and *B*). The patient was treated with a steroid taper and triamcinolone cream. The patient continued treatment with the STAT3 inhibitor while she was treated with oral steroids and topical triamcinolone. At her oncology follow-up appointment 19 days after her rash development, there was resolution of her eruption.

### Patient 2

**Fig 3:**
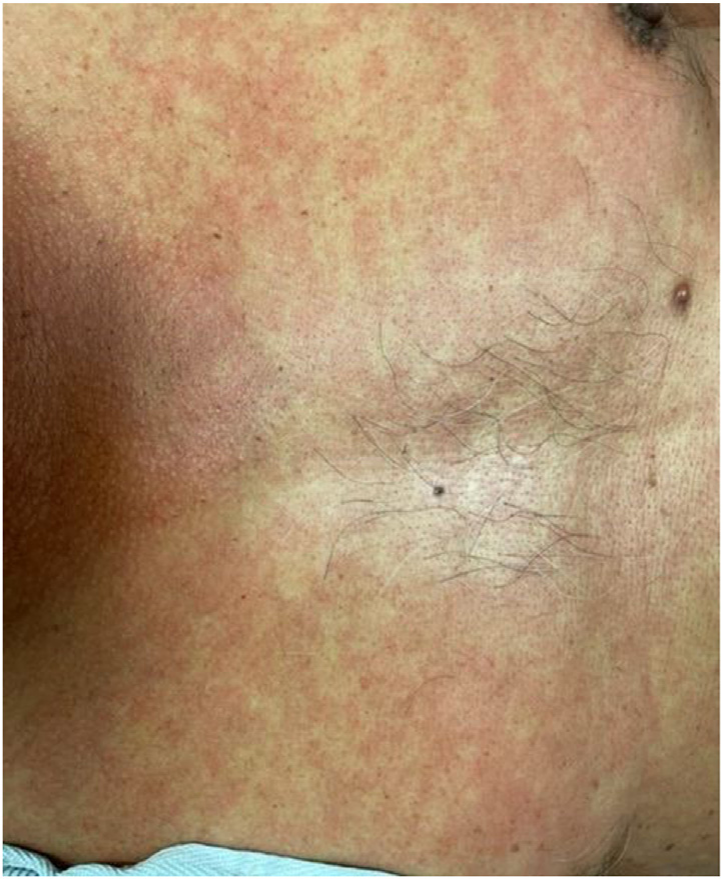
Erythematous macules on the upper chest of patient 2.

A 74-year-old male with metastatic HCC developed a grade 3 morbilliform eruption on his trunk and extremities 10 days after starting STAT3 inhibitor treatment (Fig 3). The patient was seen by dermatology and prescribed triamcinolone cream. The patient discontinued his treatment with STAT3 inhibitor due to the morbilliform eruption. At his oncology follow-up 11 days after the rash developed, the patient’s drug eruption had resolved. The patient was not rechallenged with the STAT3 inhibitor.

### Patient 3

**Fig 4:**
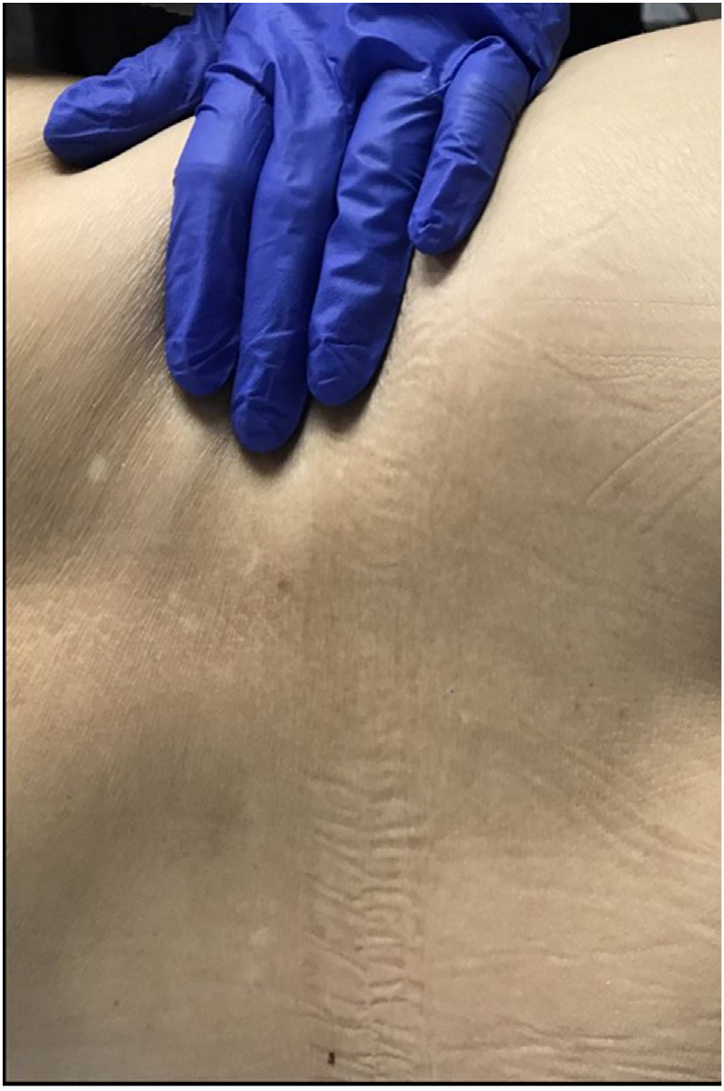
Hypopigmented macules on the lower back of patient 3.

A 66-year-old male with metastatic HCC developed asymptomatic hypopigmented patches on his abdomen and back 83 days after starting STAT3 inhibitor treatment (Fig 4). The patient saw dermatology and declined treatment as the lesions were asymptomatic. The patient was not seen by dermatology in follow-up and subsequent oncology notes do not mention his skin lesions.

### Patient 4

**Fig 5:**
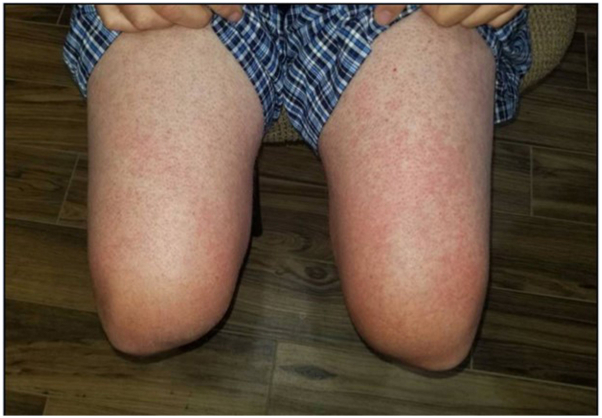
Erythematous macules on the anterior right thigh of patient 4.

A 56-year-old male with metastatic HCC developed a grade 1 morbilliform eruption (determined by review of electronic medical record photographs) 8 days after initiating STAT3 inhibitor treatment (Fig 5). The patient was not seen by dermatology. He was prescribed triamcinolone cream by his oncology team. The patient continued to receive the STAT3 inhibitor for 5 days after the start of the skin eruption. He was then found to have disease progression, and the STAT3 inhibitor was discontinued. At his oncology follow-up 5 days after the rash development, the drug eruption had resolved.

### Patient 5

A 74-year-old male with metastatic HCC developed a grade 1 cutaneous eruption as determined by his oncology team 56 days after starting STAT3 inhibitor treatment. The patient was not seen by dermatology. He was prescribed triamcinolone cream by his oncology team. 2 weeks after his drug eruption, the patient was noted to have continued skin lesions at his final oncology follow-up. The patient was not seen at our institution again.

### Patient 6

A 58-year-old male with metastatic HCC developed a grade 2 cutaneous eruption as determined by his oncology team 66 days after starting STAT3 inhibitor treatment. The patient was not seen by dermatology. He was prescribed triamcinolone cream by his oncology team. 2 months after his cutaneous eruption, the patient continued to have ongoing skin lesions seen at his final oncology follow-up prior to his death.

### Patient 7

A 55-year-old female with metastatic breast cancer who was hormone receptor-positive and human epidermal growth factor receptor 2-negative developed scaly erythematous plaques on the scalp, neck, and extremities approximately 83 days after starting STAT3 inhibitor treatment. The patient was seen by dermatology and prescribed triamcinolone ointment and fluocinonide for a suspected psoriasiform eruption. At her follow-up with oncology 30 days after her skin eruption, she had resolution of her skin lesions.

### Patient 8

**Fig 6:**
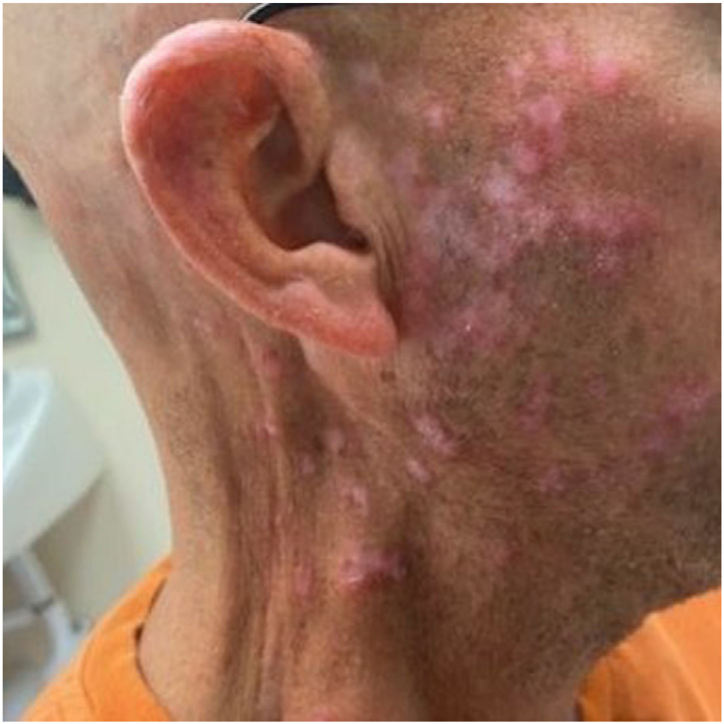
Pruritic erythematous papules on the face and neck of patient 8.

**Fig 7:**
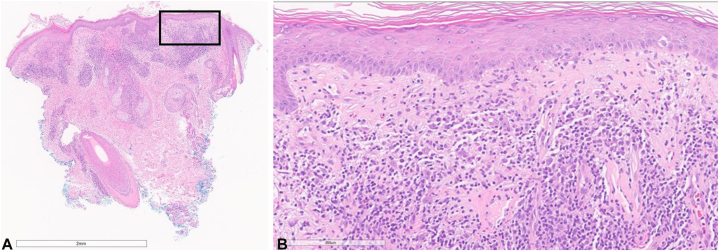
Hematoxylin-eosin stains showing lichenoid lymphohistiocytic infiltrate from patient 8. **A,** Original magnification: ×2. **B,** Original magnification: ×20.

A 46-year-old male with metastatic melanoma developed pruritic firm erythematous papules on the face and bilateral neck 25 days after starting STAT3 inhibitor treatment (Fig 6). The patient was seen by dermatology, and a punch biopsy from the right neck revealed a lichenoid and superficial perivascular dermatitis (Fig 7). After 18 months of treatment, the lesions resolved with acitretin 10 mg daily, intralesional triamcinolone 10 mg/ml, betamethasone cream, and desonide ointment. The patient continues to follow-up with dermatology and remains on the STAT3 inhibitor.

## Discussion

In this case series, we presented 8 patients with variable cutaneous reactions to STAT3 inhibitors, including morbilliform, leukodermic, psoriasiform, and lichenoid eruptions. 6 (75%) of the 8 patients had HCC, 1 had breast cancer, and 1 patient had skin melanoma. 7 of the 8 patients continued on the STAT3 inhibitor with topical and oral steroids and acitretin, with the exception of patient 2 who discontinued the STAT3 inhibitor treatment owing to skin eruption.

Determining the efficacy of the treatments was limited by the lack of dermatology follow-up and discontinuation of STAT3 inhibitors due to disease progression. Although many patients were taking multiple concomitant medications in addition to the STAT3 inhibitor, the timing of the rash and initiation time frame of all the medications in each patient’s case strongly pointed to the STAT3 inhibitor as at least a contributing factor to cutaneous toxicities. However, STAT3 inhibitors are reported to have an immunomodulatory effect, and therefore, they are expected to be associated with cutaneous complications.^2^^,^^9^

Cutaneous reactions secondary to STAT3 inhibition were less expected given the Janus kinase (JAK)-STAT pathway and the role of JAK inhibition as an important therapeutic tool in many dermatologic inflammatory conditions, including psoriasis and atopic dermatitis.^10^ JAKs and STATs are closely intertwined, as STAT3 is located directly downstream of JAKs in the signaling pathway. JAKs mediate the tyrosine-phosphorylation of STAT3, leading to STAT3 dimerization and modulation of gene transcription.^11^ JAK inhibitors are considered indirect inhibitors of STAT3 and are generally not associated with cutaneous toxicities. JAK inhibitors are primarily used for inflammatory and myeloproliferative conditions. Since the JAK/STAT3 pathway is hyperactivated in many patients with cancer, JAK inhibitors have been shown in preclinical studies to inhibit the growth of many solid tumors.^3^^,^^12^ The interplay between JAKs and STAT3 will continue to be of great interest in dermatology and oncology.

While our study was limited by the number of patients undergoing STAT3 inhibitor treatment, it is important to report cutaneous toxicities associated with STAT3 inhibitors. In our study, we found that 15.4% of patients developed a cutaneous reaction to STAT3 inhibition. As STAT3 inhibitors are increasingly used for cancer therapy and other conditions such as fibrosis^13^ or colitis,^14^ describing and managing their dermatologic adverse effects will become even more essential. Keeping in mind that the attribution of an adverse event to a drug is an exclusion diagnosis, that is, all other possible contributing factors must be excluded, it is likely that STAT3 inhibition has contributed to the cutaneous abnormalities of the patients in this report. This is particularly notable for patients 5 and 6, who developed new eruptions that started 3 to 4 months after initiation of the STAT3 inhibitors. In addition, there was no morphologic description of the eruption available. However, the treating oncologist had attributed the skin rash to the STAT3 inhibitors. These cases should be considered with caution.

This study was also limited since many of the patients’ findings were from nondermatology providers and without histologic analysis. Further studies with larger sample sizes and long-term follow-up are needed to improve the understanding of these cutaneous reactions and enhance patient care.

## Conflicts of interest

Dr Tsimberidou declares receipt of Clinical Trial Research Funding (received through the institution): OBI Pharma, Agenus, Vividion, Macrogenics, AbbVie, IMMATICS, Novocure, Tachyon, Parker Institute for Cancer Immunotherapy, Tempus, and Tvardi; fees for consulting or advisory roles for Avstera Therapeutics, Bioeclipse, BrYet, Diaccurate, Macrogenics, NEX-I, and VinceRx. Drs Lee, Chakraborty, and Patel have no conflicts of interest to declare.
